# Fellowships, Grants, & Awards

**Published:** 2006-12

## Environmental Health Sciences Core Center Grants (P30)

The National Institute of Environmental Health Sciences (NIEHS) invites applications from qualified institutions for support of the Environmental Health Sciences Core Centers. These centers are designed to build infrastructure in the field of environmental health sciences, including those needed to conduct basic, translational, clinical, and public health research. By facilitating the use of shared research resources that serve the research in the mission areas of the NIEHS, investigators who are associated with EHS Core Centers will be poised to lead the field in new and important directions. The mission of NIEHS is to improve human health by using environmental sciences to understand human biology and human disease.

A P30 Core Center grant is an institutional award, made in the name of a principal investigator, to bring together multidisciplinary groups of scientist to identify scientific opportunities and tackle compelling problems in environmental health sciences. By supporting centralized resources and facilities and fostering scientific exchange, new technologies and approaches can be brought to bear on new and existing research projects. The P30 Core Center grant is awarded competitively, initially for up to 4 years, and may be renewed for periods of up to 5 years. By providing a center structure and core resources, this support is intended to enhance the productivity of traditional research grants at the institution, focus investigators on environmental science issues relevant to human biology, human disease, and public health, and thereby improve the health of communities and the nation. A core center grant helps to integrate and promote research in existing projects and provides an administrative framework within one or several central themes; however, no funds are provided for direct support of research projects, except for pilot projects, recruitment of select new investigators, and research program development.

The core centers are expected to bring their efforts to identify promising opportunities for collaboration and support which would translate environmental health research and related basic science into domains that enhance our understanding of human disease and public health. The core center, thus, is charged with recognizing unique opportunities and capitalizing on them to foster scientific excellence using new methods, technologies, and novel scientific approaches that focus on using environmental science to understand human biology and human disease. The emphasis should be on fostering scientific excellence by providing resources and scientific interactions unlikely to be attained by individual investigators, promoting collaborations among basic biomedical and applied researchers, reaching out to innovative investigators in complementary fields, and facilitating cutting-edge research that addresses exposures and health issues in a timely manner.

In addition to direct research support services, the center should stimulate career development for future research leaders in environmental health sciences. This can include training and mentoring junior faculty in environmental health sciences, promoting interactions with established investigators in related disciplines, and helping young scientists and clinician-scientists to build foundations for careers in NIEHS-sponsored research. Investigators and trainees are encouraged to interact with NIEHS program officials with the goal of promoting grantsmanship and eventual funding by NIEHS. NIEHS strongly encourages training and career development of women and underrepresented minorities.

Overall NIEHS expects that an EHS Core Center will: 1) provide intellectual leadership and innovation in basic science, translational and clinical sciences, and public health research in environmental health; 2) stimulate integration of basic and applied research to better understand the impact of environmental exposures on human disease; 3) facilitate and develop new interdisciplinary research strategies to advance the field; 4) incorporate novel technologies and methods into EHS research; 5) provide career development for future research leaders. Membership in the center should help build identity in basic biology, translational research, clinical research, and public health through mentoring, training, and interactions with programs developed by NIEHS; and 6) stimulate and support new ideas and collaborations on topics of unique scientific opportunity that are relevant to environmental health sciences.

NIH defines human clinical research as: 1) patient-oriented research. Research conducted with human subjects (or on material of human origin such as tissues, specimens, and cognitive phenomena) for which an investigator (or colleague) directly interacts with human subjects. Excluded from this definition are *in vitro* studies that use human tissues that cannot be linked to a living individual. Patient-oriented research includes: *a*) mechanisms of human disease, *b*) therapeutic interventions, *c*) clinical trials, or *d*) development of new technologies; 2) epidemiologic and behavioral studies; 3) outcomes research and health services research.

NIEHS defines translational research as efforts that transform scientific discoveries arising from laboratory, clinical, or population studies into clinical or population-based applications to reduce disease incidence, morbidity, and mortality.

The overall goal of the EHS Core Center is to enhance the capabilities of existing programs in environmental health sciences, to assist with building the programmatic and scientific capacity for environmental health sciences, and to support the development of future directions and future leaders needed for the field to mature. The EHS Core Center must be an identifiable organizational unit within a single university, medical center, or a consortium of cooperating institutions with a university affiliation. The EHS Core Center grant mechanism provides core support to foster integration, coordination, and interdisciplinary cooperation among a group of established investigators conducting high-quality research clearly related to the effects of environmental factors on human health. The NIEHS uses this mechanism to integrate and build upon existing programs and institutional resources such as university-wide facilities and services that encourage and enhance research on environmentally induced disorders. Although the EHS Core Center grant provides support for core resources and facilities to be used by center investigators, it does not provide direct funding for ongoing research projects, but does provide limited funds for pilot projects and support for recruitment and career development of promising and outstanding investigators in environmental health sciences.

To qualify for an EHS Core Center, the applicant institution must already have an identity in environmental health sciences as defined as a substantial base of ongoing, independently supported, peer-reviewed research projects clearly dedicated to the study of environmental health sciences or environmental medicine, a substantial portion of which should be supported by NIEHS. This currently funded research base provides the major support for a group of investigators who would benefit from shared resources. The research base must exist before the submission of an application and will be a critical element considered during the peer review process. Focus, relevance, interrelationships, quality, productivity, and, to some extent, quantity, are all considerations in judging the adequacy of the research base.

Required components are: 1) The center director is the designated leader of the EHS Core Center and provides scientific and administrative leadership for the total program. The center director is required to commit a minimum of 20% effort to the center. 2) An administrative core oversees organizational, budgeting, and reporting aspects, and provides the leadership for scientific and programmatic activities of the EHS Core Center. 3) A Pilot Projects Program is required and is considered to be an integral part of the support provided. This program provides modest support for new initiatives or feasibility projects for either new investigators or for established investigators who are moving into research areas of direct interest to the EHS Core Centers. Up to 25% of the budget is to be allocated to the Pilot Projects Program. 4) Facility cores are the major function of the EHS Core Center. Cores are shared facilities that enhance research or improve cost effectiveness of services, techniques, or instrumentation used by the member investigators. Cores should extend, support, and contribute to the work of the center members. The facility cores are expected to be dynamic and respond to the needs of EHS core investigators as these needs evolve. A center should have a minimum of two facility cores including the required Integrative Health Sciences Facility Core (IHSFC; see below) and must serve at least three users.

The IHSFC is intended to facilitate translational and clinical investigations, either patient-oriented or population-based research that enhances translation of basic research findings into practical impacts for patients and communities. Services available through this core may, for example, provide the opportunity for center members to obtain clinical samples and patient data needed for their research. These services could also be directed at studies of the etiology, pathogenesis, and prognosis of disease in patient populations. 5) A Community Outreach and Education Core (COEC) is optional and serves as a source of information and expertise to the surrounding communities and stakeholders to further scientific collaborations and dissemination of research results. Inclusion of a COEC allows the center to request an additional $100,000 annual direct costs.

Applications may include up to 10% of the budget dedicated to a director’s fund to be used for rapid responses to new and emerging opportunities in environmental health sciences. As of 1 September 2005, the following changes took effect for new and competing applications to the Environmental Heath Sciences Core Centers: 1) NIEHS merged the NIEHS Core Centers and Marine Freshwater Biology Centers programs into a single program entitled The Environmental Health Sciences Core Centers Program (EHS Core Centers). 2) Site visits are no longer conducted as part of the review process. Program staff may decide to visit selected applicants to gain further information on which to base funding decisions. 3) The program endeavors to focus investigators to a greater extent on clinical applications, translation, and interdisciplinary research that will have a greater impact on human disease and public health. 4) To provide increased flexibility in organization and structure of the EHS Core Center, the director may develop a dynamic structure which meets the ongoing intellectual needs of the center. This structure can change as the intellectual needs change to accommodate new opportunities for collaboration. Research Cores are no longer required as organizational units in the center. The proposed center organization must include the required components outlined above, but, beyond those, no additional structure is imposed by NIEHS. 5) An IHSFC is required. 6) COECs, which focus on partnering with stakeholders to disseminate EHS Center research results, are optional. Centers that choose to develop a COEC are eligible for an additional $100,000 direct costs. Kindergarten–Grade 12 (K–12) curriculum development is not allowed as a COEC activity. 7) Page limits apply to the application (see Section IV, Part 6 “Other Submission Requirements” of this FOA). Applicants can download preformatted tables to facilitate completion of the application from the NIEHS website at http://www.niehs.nih.gov/centers/appguide.htm.

A vision, theme, and set of goals must be developed and described in the application. The center director must provide a written strategy for how the center will implement this vision and which future directions will likely be followed during the project period. The plan will outline the existing skills, technologies, and scientific research base and other resources at an institution. This plan should describe how the core center will enhance ongoing projects, help introduce outstanding new projects, respond to future challenges and opportunities, and promote collaborations, advances in technology, and progress in environmental health sciences. The center director must detail expected scientific outcomes including a description of anticipated impact on human disease and public health. An organizational chart should be included to illustrate the structure, interactions, and leaders of the core center. The application must define, in this section, the eligibility criteria for center membership and note which individuals play key leadership roles in the center.

This plan must address the following critical elements: 1) Theme: Provide the central theme(s) of the EHS Core Center and the likely supported research, resources, and relevance to environmental medicine. The theme may be broad or focused, depending upon the goals of the core center. 2) Goals and directions: Describe current and future directions for the core center in the forthcoming project period. How will the research supported by the EHS Center affect the understanding of environmental health sciences and, ultimately, public health? Describe the short-, mid-, and long-term goals and measures of success. What are the likely advances expected in the field of environmental health, and how can these advances be applied to human disease and public health? Describe any basic science work that has successfully been translated to the bedside or community or plans to enhance that translation in the next project period. What expected, widely applicable research tools and scientific advances will emerge from the center’s emphasis? Document how the center will organize and lead the team towards these advances. Identify levels of risk for these goals, potential roadblocks to achieving them, and how the center might respond to these challenges. Competing continuation applications must also describe the accomplishments of the center in the preceding project period and how it intends to build upon its successes. These accomplishments should be presented in three areas: basic science, clinical research, and public health science. The impact of center-based science should be discussed in detail. 3) Integration of investigators of multiple skills and talents: Outline steps the center will take to promote interdisciplinary studies and collaborations, especially among basic scientists and clinical researchers. What types of initiatives will stimulate the teams and attract high-caliber professionals? To what degree will high-risk/high-payoff research that may require long-term support be implemented? 4) Building research capacity: Provide details on the special talents and resources that will be drawn to and built upon at the center. How will these talents be harnessed and used to promote new collaborations and produce multidimensional teams to address more complex questions? Include a plan for bringing investigators into the center from within and outside EHS. Describe academic and research partnerships that will be pursued by the center to advance its goals and missions. 5) Provide a plan to determine the need for services and instrumentation of the center: Address the steps that will ensure that the core center proceeds at the cutting edge of technology and concepts. It is expected that facility cores needs may change with time. Include information on the process of re-evaluation of needs and implementation of changes. 6) Provide a plan to evaluate new and emerging opportunities in environmental health sciences and describe how the director’s fund and other center resources (facility cores and pilot project) will be redeployed to capitalize on these exciting research programs. 7) Research cores, if included, are to be discussed within the strategic vision. Brief examples of ongoing or planned research should be discussed as appropriate with reference to the supporting facility core. Do not provide an exhaustive list of ongoing incremental research. Weave significant findings and advances throughout the narrative of this section to demonstrate the leadership and impact of the center on building its environmental health sciences program. 8) Plans for a COEC (if included) must indicate how this entity will integrate with the center and fulfill its mission with the targeted audience(s).

The EHS Core Center grant mechanism fosters interdisciplinary cooperation among established investigators conducting high-quality research in environmental health science. Therefore, existence of a strong research capability in environmental health sciences is fundamental to establishment of a new, or continuation of an existing, EHS Core Center. To qualify for EHS Core Center support, an institution must demonstrate this research capability so as to have a clearly identifiable, major scientific focus in environmental health research. Consequently, an existing program of excellence in biomedical research in the field of environmental health science is a basic prerequisite for establishment of an EHS Core Center. Furthermore, a center must be able to capitalize on these research capabilities and resources to advance significantly our understanding of its chosen scientific focus.

At the time of submission of a new or competing continuation application, any institution or consortium wishing to qualify for the EHS Core Center grant must have a minimum of three active NIEHS-supported research grants from three distinct principal investigators. Acceptable grant support includes R01, R21, R37, P01, P42, P50, Cooperative Agreements (U-grants), or Research Career Development Awards (K-grants), not including administrative extensions, either with or without additional funds. Each multicomponent (e.g., P01, P50, or U01) award will count as one qualifying research project. A subproject of a multicomponent award (e.g., P01) that is subcontracted to the applicant institution can be counted only once towards the research base.

Research grant support from NIH and sources other than PHS should be listed and will be considered in the determination of its suitability of focus on environmental health sciences if the research is 1) related to human health in areas where there is evidence for the involvement of environmental factors in disease etiology or phenotypic expression; 2) of outstanding quality; and 3) funded by an entity using peer or internal review of rigor comparable to that of PHS. NIEHS will have the final decision in determining whether the applicant Center Institution has the critical mass of direct costs, grants, and investigators. Before submission of an application, the proposed center director must consult with institute staff regarding the adequacy of the research base.

Applicants must detail grants and funding sources in this section by completing, for example, Table A: Grant Support (http://www.niehs.nih.gov/centers/appguide.htm) and by describing environmental health sciences research at the applicant institution(s) that emphasize the focus, interactions, relationships, and scientific excellence of the projects and investigators and the impact on advancing scientific knowledge relevant to environmental health issues. Include in the appendix a brief abstract of approximately one-half of a page for each project.

Competing continuation applications need to describe how the existing center facilitated a leading role in environmental health at its home and associated institutions and should document the outcomes and impact of the core center on research efforts during the preceding funding period. This should include a summary of research highlights that were accomplished as a result of center infrastructure and support, how facilities were made available to the maximum number of qualified investigators, the changes in resources that might have been made to accommodate altered user needs and/or increased demand, a composite list of publications, examples of subsequent funding for new directions highlighting collaborations fostered by the center, and career advances and training outcomes. To assist in preparing the application, Table D1: Publications Resulting from Center Involvement and, if appropriate, Table D2: Publications Resulting from Pilot Program Funding Applicants, which have been preformatted to facilitate completion, can be downloaded from the NIEHS website at http://www.niehs.nih.gov/centers/appguide.htm. Measures of accomplishment also include pilot projects that led to NIH or other peer-reviewed research applications; new or improved tools, discoveries, or patented inventions (and documentation of the wide use of such tools); training and recruiting of new investigators who have advanced in their careers in environmental health; and, where applicable, outreach to affected communities and appropriate educational outcomes.

Each applicant institution will specify an experienced and respected center director with authority to oversee the organization and operation of the center and to provide scientific and administrative leadership for the total program. The center director should devote at least 20% total effort to the center. A deputy center director must also be designated to serve in the absence of the director, with other responsibilities described. The background and scientific and administrative expertise of the center director and the deputy director should be described fully in the application. For competing applications, an assessment of past performance is required.

Emphasis on career development for environmental health scientists is strongly encouraged. The application should address plans that will promote training of new investigators and bring new expertise into the area of environmental health sciences by supporting the career development of established investigators. Specify the plans to cross-train researchers in current techniques that are absent from the EHS Core Center or individual research programs. Training and cross-training may include collaborations that will introduce a focus on human subjects and tissues into laboratory-based studies. NIEHS strongly encourages training and career development of women and underrepresented minorities.

The following activities are consistent with this aspect of the EHS Core Center: 1) Temporary salary support (up to 75%) and equipment may be provided in the application for a Named New Investigator in a specified area of research. The investigator can be a worker in the basic sciences, clinical research, or public health disciplines relevant to environmental health. Former postdoctoral assistants and fellows are eligible for this position.

This investigator is eligible to compete for support for up to 2 years through the pilot project program. Subsequently recruited individuals are to be named by the center director and submitted for approval to the Center's Internal or External Advisory Board, as appropriate. 2) The EHS Core Center grant may provide partial salary support (up to 75%), technical support, and equipment for independent investigators newly recruited from outside the center. This mechanism is intended to infuse center research with novel technologies and approaches by supporting independent investigators, ideally, who are at the beginning stages of their research careers, and will add needed expertise to the center structure. The recruit would be expected to bring new technologies or novel scientific areas of expertise into the environmental health sciences arena that enhance the center’s research capabilities. Former graduate students and postdoctoral fellows of center members should not be considered for support unless, in exceptional cases, it can be demonstrated that they have established independent research careers and will provide critical expertise. Funds awarded under this section may be used for salary, technical support, and equipment. The remaining salary support for the recruited center investigator must be derived from other than center funds. For each investigator, the duration of support as a recruited center investigator will be limited to no more than 2 years. Specific individuals to be awarded recruited center investigator support need not be identified in the application, but the amount budgeted for this purpose should be declared, and, to the extent possible, the types of individuals sought and their expected roles in the center described. Competing continuation applications should include a discussion of how these funds were used in the previous project period in terms of who was recruited and how these individuals benefit the center programs.

The EHS Core Centers mechanism requires clinical and basic scientists with a broad range of skills to work together on a unified theme. Therefore, it presents a rich environment for young translational and clinical investigators to be exposed to and develop additional research skills. Mid-level investigators and scientists in other fields may also be attracted by opportunities in the center to focus their attention on issues in environmental health sciences and human disease. Financial support can be provided for training and mentoring of physician scientists to study environmental health issues that are relevant to in translational and clinical research or public health. In addition, environmental health scientists can be supported to engage in activities which increase their understanding of clinical research. The objective of this activity would be to assist new investigators in progressing to more senior status and eventual NIEHS funding by enhancing their research skills and knowledge in translational and clinical research. These activities can be constituted as an independent facility core, or as part of the administrative core.

The career development activities should be directed by an investigator with strong mentoring credentials who will devote a defined percent effort (5% suggested). To facilitate mentoring and multi-disciplinary developmental activities, active involvement by senior investigators within the core center is strongly encouraged in an effort to match mentors with candidates. The plan for career development activities will be evaluated in terms of potential effectiveness in developing the skills and research capabilities of investigators as reflected in the following required elements of the application: 1) a discussion of how mentoring and the professional development of the investigators will be achieved, including their progression to a more independent status; 2) a plan for monitoring the progress of the career development of selected investigators; 3) examples of planned scientific enrichment activities for selected investigators including training experiences, mini-sabbaticals, special lectures, visiting scientist symposia, seminars, workshops, and short courses both at the parent institution or off-site.

To increase diversity in the student and faculty populations and the participation of individuals currently underrepresented in the biomedical, clinical, behavioral, and social sciences, applicants are encouraged to designate new and recruited investigators from the following groups: women; underrepresented racial and ethnic groups; individuals with disabilities; and individuals from socially, culturally, economically, or educationally disadvantaged backgrounds that have inhibited their ability to pursue a career in health-related research.

Direct costs for the sum of career development activities should not exceed $150,000 annually. Assisting new investigators in attaining independent status or established investigators in developing new promising areas of expertise should be an objective of the Core activities. Sponsored participants should be encouraged to apply for NIEHS sponsored Career Development Awards, patient-oriented research grants, or other types of independent support. Contact with NIEHS program staff is encouraged at an early stage in submission of new applications.

The institutional commitment at the applicant institution will be a major consideration in ensuring the goals of the Core Center. The parent institution should recognize the EHS Core Center as a formal organizational component and provide documented evidence of space dedicated to the needs of center, protected time to devote to center activities, staff recruitment, dedicated equipment, or other financial support for the proposed center. The parent institution should provide assurance of its commitment to continuing support of the EHS Core Center in the event of a change in directorship and a well-defined plan for this eventuality should be in place. In addition, it is expected that the institution will support the goal of providing to center members priority access to institution’s and center’s facilities and services at minimal cost.

The organization and structure of the EHS Core Center should reflect the goals of the center, encourage collaboration, develop and implement center-wide initiatives, and promote the use of shared resources and pilot project funds. The structure can change as needed based on new scientific opportunities and partnerships. This is a major conceptual change in the EHS Core Centers that will allow modification of programmatic and scientific activities and areas of support to fully capitalize on the most exciting research opportunities in environmental health sciences.

The application should include a description of the organization and structure of the center and illustrate all components in an organizational chart.

It is expected that organization of the administrative core will provide a supportive structure sufficient to ensure accomplishment of the following: 1) coordination and integration of center components and activities; 2) assessment of productivity, effectiveness, and appropriateness of center activities and determination of center membership assessment of scientific opportunities and areas for collaboration among center members; 3) organization of center activities, such as retreats, invitation of consultants, meetings, and focus groups; 4) organization of the Internal and External Advisory Groups; 5) record keeping of meeting minutes and measures of success, including use of EHS Core Center facilities, publications, pilot project awards, and new grant applications resulting from preliminary data enabled by the center; 6) interactions with other centers, the NIEHS, and other appropriate individuals, groups, or organizations.

The administrative structure must include an Internal Advisory Committee (IAC) and an External Advisory Committee (EAC). Further details for constitution of these committees are available in the complete set of Guidelines for Environmental Health Sciences Core Center Grants (http://www.niehs.nih.gov/centers/appguide.htm; Updated September 1, 2005).

Competing continuation applications must document the functions and effectiveness of the external and internal advisory committees.

The administrative core may include a director’s fund, with up to 10% of a center’s annual budget, to be used in rapid response to special needs and to exploit emerging scientific advances and novel opportunities. Examples of such opportunities include but are not limited to recruiting additional investigators or engaging collaborators with needed expertise; fostering translation of advances to the human disease or public health arena; updating equipment to improve the available technology and thereby enhancing productivity and scientific discovery; responding to unforeseen events with a direct environmental health connection. A process for expending the director’s fund should be briefly described in the application and input should be sought from the center's governing boards and external advisors when making allocations. Decisions regarding the director’s fund and subsequent outcomes should be described in annual progress reports.

To assist in preparing the application, Table B: Center Members, which has been preformatted to facilitate completion, can be downloaded from the NIEHS website at http://www.niehs.nih.gov/centers/appguide.htm and inserted into this section.

The major function of the center grant is to support facility cores that are designed to furnish groups of center investigators with techniques, services, or instrumentation that will enhance the research in progress, consolidate manpower effort, and contribute to cost effectiveness. At least three investigators with independently funded projects and demonstrated need for such a core service form the minimum required research base to establish a core facility. Additionally, the minimum of three funded investigator users does not in itself provide sufficient justification for establishment of a facility core. The center must have at least two facility cores. A new requirement is the IHSFC, which is described, below. To assist in preparing the application, Table C: Facility Core Use, which has been preformatted to facilitate completion, is provided and can be downloaded from the NIEHS website at http://www.niehs.nih.gov/centers/appguide.htm. Separate Tables, such as Table C, are to be provided for each facility core.

Facility cores should draw on center research needs, including but not limited to animal use and transgenics, imaging, tissue culture, pathology support, statistical support, oligonucleotide synthesis, analytical chemistry, proteomics, bioinformatics, exposure assessment, and handling of human tissue specimens. Establishment and continued support for facility cores by an EHS Core Center application must be justified on the basis of use by independently funded center investigators. The use of facility cores by pilot projects is encouraged. Use of core facilities by projects funded by research and development contracts will be evaluated on an individual basis. In general, use of core facilities by contracts must be paid in full from the contract funds, not from the EHS Core Center grant funds.

Facility cores for the EHS Core Center should be unique and are not to duplicate services or facilities that already exist at the parent or collaborating institutions or can be purchased commercially. University-wide facility cores providing services in areas relevant to environmental health research have become more widely available at many research centers. EHS Core Centers should use existing facility cores where appropriate and describe in the application how members of the EHS Core Center would receive priority access, favorable cost arrangements, and training on unique technologies. If facilities within a university-wide facility are not sufficient to meet the needs of the EHS Core Center, then the applicant is to provide information on the existing facilities and on how the center and greater university facility plan to partner. Proposed center facility cores that appear to replicate services already available at the applicant institution will not be allowed without extensive justification. Facility cores should not duplicate services that can be purchased in the private sector at prices below university-derived costs.

The application must provide the total operating budget for each facility core together with the percentage of support requested from the center grant. User logs or similar information used to complete the online form should be maintained and made available on request to the NIEHS to validate the extent of use and degree of sharing. In the case of new proposed centers or new facility cores within an existing center, similar information regarding anticipated use of the cores should be provided. Define the use or expected use of the facility core by center members and/or projects in terms of low, medium, or high (on a scale of 1–3).

Each facility core must have a designated leader who will be responsible for core activities. The application should explain the organization and proposed mode of operation of each core. It should include a plan for prioritizing investigator use of the core as well as a definition of qualified proposed and potential users. This definition need not be too narrow, since limited use of a core might be an enticement to established investigators in other fields to lend their expertise to the field of environmental health. The use of the facility core for training purposes is encouraged, and, if so planned, a description of the extent of and approach to this training should be included.

Although facility cores are meant to provide services for center members, they also play an important role in developing new methodologies, adapting instrumentation for center needs, and educating center members of the value and utility of services and methods. Limited funds can be designated to support these aspects of the facility cores and discussion of how these activities will be performed should be included in the application.

The IHSFC is required and should be designed to facilitate the translation of basic research findings into clinical or public health applications. This core provides new and critical resources and will be a vital component of the progression of environmental health sciences from the bench to the bedside and to affected communities. It is expected that the concepts and goals of environmental medicine will be integrated into the range of activities that the greater core center undertakes.

This core is to be designed to support collaborative efforts among basic scientists, clinical researchers, and/or public health practitioners by 1) providing services and access to instrumentation and technologies that foster integration of basic science, public health research including epidemiology and intervention studies, and patient-oriented clinical research; 2) supporting research to improve early detection, prevention, and/or therapy for environmentally-related disorders; 3) enhancing partnerships between researchers and community based organizations that affect conduct of clinical and public health research.

Among its functions, the IHSFC may provide services that capitalize on access to well-characterized patients and control subjects for research projects. These can include study subject recruitment and retention activities, and follow-up by mail, phone, or in person to gather needed data for research projects. Clinical services may include clinical laboratory or other assessments, pathology services, collection, processing and long-term storage of human tissue samples, blood, urine, or other biospecimens, and preparation of questionnaires or other assessment tools. The IHSFC can facilitate and support partnerships between study investigators and human populations or communities, health care providers or others. Description of services, equipment, and other activities of this core need to be well documented. When applicable, procedures for collecting, storing, and distributing biological samples, should be included in the application. Partnerships with other units at the institution that support these types of activities (e.g., clinical and translational science awards) are encouraged and letters of support should be included in the application. As for all facility cores, the application should include a description of the types of research projects and/or clinical trials that use or plan to use the core. Include specific examples and the likely benefits to other research activities.

For the purposes of the EHS Core Centers, clinical research is as defined by NIH. This definition can be found in the Guidelines for Environmental Health Sciences Core Center Grants at http://www.niehs.nih.gov/centers/appguide.htm.

Inclusion of a Pilot Projects Program is required and is an integral part of the EHS Core Centers. A plan to support pilot studies for basic or clinical biomedical, epidemiologic, educational, or behavioral research should be included and budgeted in the application. The description of a plan to solicit, review, and administer pilot grants must be included in the administrative core and a separate budget, including the total request for pilots, must be submitted. Criteria for review of pilot studies must be developed and included in the application. Up to 25% of the direct cost budget for each year should be allocated to the Center Pilot Projects Program to support short-term projects to explore the feasibility of new areas of study which leads to collection of sufficient data to pursue support through other funding mechanisms. Include a clear description of the process designed to award and evaluate progress in pilot projects. Investigators are encouraged to consult with NIEHS program staff for submission of new NIH applications based on pilot project-supported data.

Competing continuation applications should provide documentation of the existing pilot projects program. Include the process for application review and award and the measures of success, such as publications, subsequent funding, and career advancement of the sponsored individuals. A competing continuation application should include: historical overview of the Pilot Project Program during the last program period; a description of the management of the program; and a listing of all pilot projects which were supported during the last project period. To assist in preparing the application, Tables E1: Pilot Projects Outcomes and E2: Grant Details for Pilot Projects, which have been preformatted to facilitate completion, are provided and can be downloaded from the NIEHS website at http://www.niehs.nih.gov/centers/appguide.htm and inserted into this section.

Pilot projects are primarily intended to 1) provide initial support for new investigators to establish new lines of research; 2) allow exploration of possible innovative new directions representing a significant departure from ongoing funded research for established investigators in environmental health sciences; ideas of particular importance in environmental health sciences are paramount; 3) stimulate investigators from other areas of endeavor to apply their expertise to environmental health research and environmental medicine; 4) foster opportunities that meet goals set out in the EHS Core Center Plan. Pilot projects should strive to fill in gaps in research areas relevant to the scientific focus of the core center.

NIEHS Core Centers have the option to develop and sustain community outreach and education activities. The objective of the COEC is the translation of research information into knowledge for various professional and public stakeholders. Therefore, each center that chooses to develop a COEC must demonstrate that the objectives, activities, and products are aligned and integrated with the research strengths and focus of their center.

Programs developed by COECs will lead the field of environmental health outreach and education at the local and national level. To this end, the goals of the COEC are to 1) develop partnerships with stakeholders to translate and disseminate EHS Core Center science; 2) work with community-based organizations, disease advocacy groups, and other local, state, or regional partners to enhance the dialogue on environmental health issues in their regions; 3) develop and implement appropriate outreach and educational programs to increase awareness and understanding of environmental health research being conducted at the EHS Core Centers; 4) evaluate outreach models, disseminate results at local and national levels, and promote models for national implementation.

To meet these goals, it is essential for COECs to state clear and measurable objectives; possess appropriate expertise to fulfill its stated objectives; identify specific environmental problems; demonstrate alignment to research strength and focus of the center; identify existing and future partners; prioritize short-, mid-, and long-term activities to be implemented; list and describe expected products; state anticipated impacts and their significance for environmental public health; and define evaluation tools to measure the impact of core activities.

For the purposes of the EHS Core Center Program, there are three target audiences of interest: community, policy makers, and public health and/or health care professionals. COECs may select more than one target audience, but are only required to choose one. Examples of activities are in the EHS Core Center Application Guidelines at http://www.niehs.nih.gov/centers/appguide.htm.

Should a core center choose to support a COEC: 1) The COEC is required to establish a Stakeholder Advisory Board to strengthen the bidirectional interaction between the core center and partners. The purpose of this advisory group is to ensure center understanding of community and other stakeholder needs, as well as to ensure more effective dissemination of center research in appropriate venues. The center should develop a specific plan and set of integrated activities for COEC, particularly with respect to the center’s defined community and target audience. COEC must be a logical outgrowth of the scientific focus of the center and exhibit the potential for mutual benefit due to interactions with center investigators. 2) COECs must possess the appropriate expertise for the identified target audience and outlined activities. It is important that COECs be directed by staff trained in public health, outreach and education, and other relevant disciplines at a master’s or doctoral level. 3) Collaborations among COECs in EHS Core Centers are desirable. Support of collaborations can be from NIEHS/NIH or other agencies and foundations. 4) COECs are encouraged to collaborate with NIEHS staff within the Division of Extramural Research and the Office of Communication and Public Liaison in developing printed and audiovisual educational materials. These outreach activities must be identified as programs supported by the NIEHS Core Center. All COEC-produced materials must be submitted to the Community Outreach Resource Center. 5) Support for appropriate staff positions, travel, equipment, and supplies for this activity is allowed. 6) Although COEC is not intended to include human subject research, epidemiology, clinical trials, clinical services delivery, or community-based research, the core may be useful in establishing a relationship with a community-based organization that could form the foundation of a research grant application. In such cases, appropriate COEC proposals may be considered for pilot project funding. The program should not go beyond public and community education concerning environmental disease risk and/or hazard exposure recognition, as the COEC is not intended to give medical, legal, political, social, or economic advice. 7) K–12 curriculum development is not allowed as a COEC activity.

This funding opportunity will use the P30 award mechanism. As an applicant, you will be solely responsible for planning, directing, and executing the proposed project.

This funding opportunity uses the just-in-time budget concepts. It also uses the nonmodular budget format described in the PHS 398 application instructions (see http://grants.nih.gov/grants/funding/phs398/phs398.html). A detailed categorical budget for the "Initial Budget Period" and the "Entire Proposed Period of Support" is to be submitted with the application.

The PHS 398 application instructions are available at http://grants.nih.gov/grants/funding/phs398/phs398.html in an interactive format. Applicants must use the currently approved version of the PHS 398. For further assistance contact GrantsInfo, 301-435-0714, (telecommunications for the hearing impaired: TTY 301-451-0088), or by e-mail: GrantsInfo@nih.gov.

Applications must be prepared using the most current PHS 398 research grant application instructions and forms. Applications must have a D&B Data Universal Numbering System (DUNS) number as the universal identifier when applying for federal grants or cooperative agreements. The D&B number can be obtained by calling 866-705-5711 or through the web site at http://www.dnb.com/us/. The D&B number should be entered on line 11 of the face page of the PHS 398 form.

The letter of intent receipt date for this RFA is 19 January 2007, with the application receipt date 21 February 2007. The complete version of the RFA is available at http://grants.nih.gov/grants/guide/rfafiles/RFA-ES-06-010.

Contact: Leslie Reinlib, Division of Extramural Research and Training, National Institute of Environmental Health Sciences, PO Box 12233 (EC-21), 79 T.W. Alexander Drive, Room 3453, Research Triangle Park, NC 27709-2233 USA, 919-541-4998, fax: 919-316-4606, e-mail: reinlib@niehs.nih.gov.

Reference RFA-ES-010

## Independent Scientist Award (K02)

The Independent Scientist Award (K02) is intended to foster the development of outstanding scientists and enable them to expand their potential to make significant contributions to their field of research. It provides 3, 4, or 5 years of salary support and “protected time” for newly independent scientists (see IC provisions) who can demonstrate the need for a period of intensive research focus as a means of enhancing their research careers. Each independent scientist career award program must be tailored to meet the individual needs of the candidate. The sponsoring institution must demonstrate a commitment to provide the environment, resources, and the protected time required for the candidate to perform the activities included in the proposed research and career development plans.

The participating NIH Institutes and Centers (ICs) have distinctive guidelines, requirements, salary and research support levels provided under the auspices of this funding opportunity announcement to accommodate the career needs of researchers working in fields related to their specific research missions. Potential applicants are strongly advised to contact the appropriate NIH staff member identified in Section VII (http://grants.nih.gov/grants/guide/contacts/pa-06-527_contacts.htm) to discuss their particular circumstances and eligibility for the K02 award before developing an application.

This funding opportunity will use the NIH K02 award mechanism. The candidate and sponsoring institution will be responsible for planning, directing, and executing the proposed project and independent scientist award activities.

This funding opportunity uses the just-in-time budget concepts. It also uses the nonmodular budget format described in the PHS 398 application instructions (see http://grants.nih.gov/grants/funding/phs398/phs398.html). The applicant should follow the instructions for budget information described in the PHS 398, Section III, providing only the total direct costs for each year and the entire proposed period of support and a budget justification.

Project periods may be for up to 5 years, at least 3 years are required. Awards may be competitively renewed at the discretion of the participating NIH ICs. Only a few of the NIH awarding components permit competitive renewals of this award; see http://grants.nih.gov/grants/guide/contacts/pa-06-527_contacts.htm.

The PHS 398 application instructions are available at http://grants.nih.gov/grants/funding/phs398/phs398.html in an interactive format. Applicants must use the currently approved version of the PHS 398.

For further assistance contact Grants Info, 301-435-0714, (telecommunications for the hearing impaired: TTY 301-451-0088), or by e-mail: GrantsInfo@nih.gov.

Applications must be prepared using the PHS 398 research grant application instructions and forms. Applications must have a Dun and Bradstreet (D&B) Data Universal Numbering System (DUNS) number as the universal identifier when applying for federal grants or cooperative agreements. The D&B number can be obtained by calling 866-705-5711 or through the web site at http://www.dunandbrad-street.com. The D&B number should be entered on line 11 of the face page of the PHS 398 form.

The application submission date for this PA are available at http://grants.nih.gov/grantsfunding/submissionschedule.htm. The complete version of this PA is available at http://grants/nih.gov/grants/guide/pa-files/PA-06-527.

Contact: Applicants should refer to the NIH web site (http://grants.nih.gov/grants/guide/contacts/pa-06-527_contacts.htm) for information regarding each IC’s scientific/research contacts for this K02 program.

Reference PA-06-527

